# Full Genome Sequence of a Novel Human Enterovirus C (EV-C118) Isolated from Two Children with Acute Otitis Media and Community-Acquired Pneumonia in Israel

**DOI:** 10.1128/genomeA.00121-12

**Published:** 2013-01-31

**Authors:** Cristina Daleno, Antonio Piralla, Alessia Scala, Fausto Baldanti, David Greenberg, Nicola Principi, Susanna Esposito, CAP-PRI Study Group

**Affiliations:** a Department of Pathophysiology and Transplantation, Pediatric Clinic 1, Università degli Studi di Milano, Fondazione Istituto di Ricovero e Cura a Carattere Scientifico (IRCCS) Ca’ Granda Ospedale Maggiore Policlinico, Milan, Italy; b Molecular Virology Unit, Fondazione IRCCS Policlinico San Matteo, Pavia, Italy; c Pediatric Infectious Disease Unit, Soroka University Medical Center, and the Faculty of Health Sciences, Ben-Gurion University of the Negev, Be’er-Sheva, Israel

## Abstract

The new enterovirus C strain EV-C118 belongs to the human enterovirus C species of the *Picornaviridae* family. We report the complete genome sequence of this strain, which was identified in respiratory specimens of two children hospitalized in Israel because of acute otitis media and community-acquired pneumonia who were enrolled in the Community-Acquired Pneumonia Pediatric Research Initiative (CAP-PRI) study.

## GENOME ANNOUNCEMENT

The genus *Enterovirus* in the family *Picornaviridae* is a group of related viruses associated with various clinical syndromes, including asymptomatic infection, respiratory illness, gastroenteritis, and meningitis (1). There are four known species of enteroviruses, which are classified on the basis of their divergences within the VP1 region: human enterovirus (HEV) types HEV-A, HEV-B, HEV-C, and HEV-D (2, 3). We report the characterization of a new HEV-C species, EV-C118, which is the 23rd identified so far (http://www.picornaviridae.com/enterovirus/ev-c/ev-c.htm).

EV-C118 was found in two Bedouin children living in Israel who were hospitalized because of acute otitis media (AOM) and community-acquired pneumonia (CAP). One strain, ISR10, was found in a 21-month-old girl and the other, ISR38, was found in a 15-month-old boy. Their nasopharyngeal samples were positive for enterovirus as revealed by PCR using primers that target the VP1 region as described previously (4). The complete genome sequences were obtained using degenerate primers designed by means of the multiple alignment of the EV-C104 and EV-C109 genomes available in GenBank, and additional primers were designed using primer-walking methods. These primer sequences are available upon request. The terminal sequences were confirmed using a 5′/3′ Rapid Amplification of cDNA Ends (RACE) kit (Roche, Mannheim, Germany). The sequences were aligned using ClustalX 2.1 (5), and the phylogenetic trees were constructed using Molecular Evolutionary Genetics Analysis (MEGA) 5 (6). The bootscan and similarity plot analyses were made using SimPlot 3.5.1 (7).

ISR10 was designated EV-C118 by the *Picornaviridae* study group on the basis of the sequencing of the complete VP1 region (GenBank accession no. JQ768163) (http://www.picornastudygroup.com/types/enterovirus/ev_c_types.htm). Its closest relative is EV-C109, which has 30.1% nucleotide and 22.6% amino acid differences in the VP1 region. The full genomes of both EV-C118 strains are 7,357 bp in length, excluding the polyadenylated tract. The genome organization of these viruses is similar to those of the previously reported EV genomes. The 5′ untranslated region (UTR) contains 666 nucleotides, and the 3′ UTR contains 70 nucleotides. EV-C118 has a single open reading frame that encodes a 2,206-amino-acid polyprotein. The base compositions of the full genomes of ISR10 and ISR38 strains are 27.9% A, 24.0% C, 24.3% G, and 23.7% U. Phylogenetic analysis showed that both strains of EV-C118 grouped with EV109 and that the recently described EV105 (8) is in a separate subgroup of HEV-C.

The genome sequences of the P2 and P3 regions of both EV-C118 strains are more similar to those of EV-C109 and EV-C105 than that of the P1 region. SimPlot analysis showed that the similarity is greatest in the 3D gene, which had a high level of bootstrap support (>75%) for clustering with EV-C105. As in the cases of EV-C104 and EV-C109, the 5′ UTR of EV-C118 is phylogenetically distinct from the classical HEV-C 5′ nontranslated region (NTR) (9, 10). This suggests a recombinant origin and, therefore, independent evolution of the different genomic regions; however, further extensive analyses are needed to determine its origin precisely.

### Nucleotide sequence accession numbers. 

The genome sequences of EV-C118 strains ISR10 and ISR38 have been deposited in GenBank under the accession no. JX961708 and JX961709.
